# Special Issue “Women’s Special Issue Series: Insects”

**DOI:** 10.3390/insects17010075

**Published:** 2026-01-09

**Authors:** Carmen Scieuzo, Rosanna Salvia, Patrizia Falabella

**Affiliations:** 1 Department of Basic and Applied Sciences, University of Basilicata, 85100 Potenza, Italy; carmen.scieuzo@unibas.it (C.S.); r.salvia@unibas.it (R.S.); 2 Spinoff XFlies s.r.l, University of Basilicata, 85100 Potenza, Italy

The scientific community has long recognized that diversity drives innovation, creativity, and progress. In recent decades, the number of women contributing to entomological research has grown steadily, enriching the field with new perspectives, methodologies, and areas of inquiry. However, the majority of professional STEM (science, technology, engineering, and mathematics) scientists are still male, despite increasing levels of female graduates in those areas. In particular, women remain markedly underrepresented in entomology positions within both academia and the federal government; despite accounting for 40–50% of doctoral graduates over the past decades, women entomologists occupy positions far below their share of degree holders and are therefore underemployed [1]. This Special Issue of *Insects* journal, titled “Women’s Special Issue Series Insects” celebrates the remarkable achievements of women who have shaped and continue to shape our understanding of insect biology, ecology, physiology, behavior, and applied entomology. By recognizing and amplifying the voices of women in entomology, this Special Issue underscores a broader message: that scientific progress thrives in an environment of equity, inclusion, and collaboration. This initiative will encourage continued efforts toward a more balanced and supportive scientific community for all. The works included in this issue showcase the breadth and depth of insect science, from fundamental studies to applied innovations, demonstrating how the contributions of women scientists have expanded the boundaries of knowledge and opened new avenues for discovery. The papers included in this Special Issue reflect the remarkable diversity of research led by women scientists in insect science. The contributions span multiple disciplines, from molecular biology and genetics, exploring gene expression, transcriptomics, and insect adaptation, to ecology and conservation, addressing species distribution, habitat connectivity, and biodiversity assessment. Several studies focus on applied entomology, highlighting innovative uses of insects in sustainable food and feed production, while others deal with medical, veterinary and forensic entomology, investigating vector-borne pathogens, host interactions, human health implications and emphasizing the integration of molecular approaches in crime scene investigations. Together, these works exemplify the breadth and scientific excellence of women-led research in entomology, demonstrating how their contributions continue to shape and expand the frontiers of insect science.

Below is an overview of the paper for each of the treated topics reported.

- **Insect physiology, genetics, and molecular biology**

Several papers in this Special Issue illustrate how women-led research is advancing our understanding of insect physiology and molecular responses to environmental and anthropogenic stress carrying out studies focusing on molecular mechanisms, gene expression, and genetic markers in insects, including RNA interference, transcriptomics, and enzymatic regulation related to adaptation or pesticide exposure.

Mysore et al. (contribution 1) explored a novel RNAi-based biocontrol strategy using engineered *Saccharomyces cerevisiae* expressing shRNA targeting *Drosophila suzukii* Rbfox1. Their work demonstrates the potential of RNAi-yeast baits for species-specific and environmentally sustainable pest control. This strategy demonstrates the feasibility of combining molecular precision with environmental safety in integrated pest management programs. At the same time, insights from molecular ecology reveal how insects naturally adapt to chemically diverse environments.

Nakano et al. (contribution 2) investigated how *Papilio memnon* larvae modulate CYP6B gene expression in response to host plant chemistry, revealing key detoxification mechanisms underlying host adaptation. Transcriptomic analyses showed that several cytochrome P450 CYP6B subtypes (particularly *CYP6B2*, *CYP6B5*, and *CYP6B6*) are differentially expressed in the larval fat body and midgut when larvae feed on different citrus species. These variations correlate with the presence of distinct phytochemicals in each host plant, suggesting that CYP6B enzymes play key roles in metabolizing plant secondary compounds (contribution 2).

In the context of pest adaptation and molecular responses to chemical stressors, Zhang et al. (contribution 3) generated a comprehensive catalog of SSR and SNP markers in *Tuta absoluta* exposed to different insecticides, providing essential genomic tools for understanding pesticide resistance and supporting precision pest management. *T. absoluta*, a major invasive pest of tomato crops, exemplifies this challenge. A recent study identified an extensive set of 25,123 SSR loci and 332,537 SNP loci from transcriptome data, offering a comprehensive genomic toolkit for this species. Most SSR-associated transcripts were linked to core cellular metabolic functions, while SNP-transcripts were enriched in pathways related to peroxisomes, RNA transport, carbon metabolism, and protein processing in the endoplasmic reticulum—biosynthetic routes closely tied to detoxification processes. These molecular pathways likely contribute to the enhanced survival and pesticide susceptibility profile of *T. absoluta*. Beyond their functional relevance, the newly identified SSR and SNP markers provide valuable resources for constructing genetic maps, assessing population diversity, and elucidating gene functions, ultimately supporting the development of more targeted and sustainable management strategies for this globally significant pest (contribution 3). These studies illustrate two complementary facets of insect–chemical interactions: on one hand, the endogenous genetic flexibility that allows insects to metabolize complex plant defenses; and on the other hand, the potential of molecular biotechnology to exploit gene silencing mechanisms for pest control. Integrating knowledge of insect molecular physiology, detoxification pathways, and RNAi-based technologies could pave the way for more selective, sustainable, and biologically informed pest management strategies.

Together, these studies highlight how women-led research in molecular entomology bridges fundamental mechanisms with applied innovations.

- **Ecology, distribution, and conservation of insects and arthropods (research exploring distribution patterns, habitat connectivity, and biodiversity of insects and related arthropods, with implications for conservation biology and landscape ecology).**

Research in this Special Issue also advances our understanding of species distributions, habitat connectivity, and biodiversity, providing in-sights essential for conservation planning.

Kiewra et al. (contribution 5) document recent range expansion of *Dermacentor reticulatus* in southwestern Poland using extensive field sampling and spatial statistics. Their findings underscore the influence of urbanization and landscape configuration on vector distribution and disease risk. Comprehensive faunistic surveys in montane ecosystems provide foundational knowledge for conservation. Investigations of Thysanoptera in the Góra Bucze Landscape-Nature Complex in the Western Carpathians (Poland) recorded 30 thrips species, including both herbivores and predators (contribution 6). Species-specific associations with monocotyledonous and dicotyledonous plants, as well as variations in abundance across meadow–pasture complexes, underscore the complexity of arthropod communities and the importance of detailed biodiversity assessments in mountainous habitats. Similarly, Ivković et al. (contribution 4) showed how freshwater macroinvertebrates such as *Ibisia marginata* demonstrate highly specific habitat preferences that influence their distribution and life cycles. Long-term monitoring in Croatia’s Plitvice Lakes National Park revealed that this univoltine species exhibits peak adult emergence in July, with larvae favoring moss and gravel substrates (contribution 4). These findings underscore the importance of understanding habitat-specific requirements for maintaining stable populations of aquatic predators. In terrestrial systems, species persistence is closely linked to habitat quality and connectivity.

Della Rocca et al. (contribution 7) demonstrated that *Saga pedo*, a parthenogenetic species, inhabits fragmented xerothermic grasslands in Italy. Spatial Bayesian modeling identified suitable habitat patches and ecological corridors, revealing that intensive cultivation reduces occurrence and connectivity, whereas open habitats with moderate woody cover support species persistence. This study highlights the critical role of landscape management in preserving both populations and functional connectivity for sensitive species.

Taken together, these studies illustrate the diverse ecological strategies and distributional dynamics of invertebrates across freshwater, terrestrial, and montane ecosystems. They emphasize the need for integrated approaches combining field surveys, long-term monitoring, and spatial modeling to inform conservation planning, habitat management, and the mitigation of anthropogenic pressures on vulnerable species.

- **Applied entomology in feed (applied studies investigating the use of insects or insect-derived products in food, feed, and industry, highlighting sustainable and innovative approaches to nutrition and environmental mitigation).**

A major theme within the Special Issue involves applied entomology addressing global challenges related to sustainable food systems.

Orkusz & Orkusz (contribution 8) evaluate the fortification of wheat bread with *Acheta domesticus* powder into wheat bread formulations. Their findings show that increasing levels of substitution led to higher protein, fat, fiber, zinc, and riboflavin contents, though accompanied by changes in crumb color, texture, and aroma (contribution 8). Sensory evaluation revealed that up to 15% inclusion achieved the optimal balance between improved nutrition and palatability, marking a feasible threshold for product development. This highlights the potential of edible insects to promote healthier and more sustainable dietary alternatives while minimizing formulation challenges. Beyond direct human consumption, insect-based biotechnology also demonstrates value in animal production. Hăbeanu et al. (contribution 9) investigated the use of the yeast *Rhodotorula glutinis* as a dietary supplement in silkworm (*Bombyx mori*) rearing, showing that supplementation of mulberry leaves with different yeast concentrations led to significant variations in productivity in two native breeds (Lines C and Z). At moderate concentrations (1 × 10^7^ CFU/mL), Line C larvae achieved higher values for key productive traits, including larval growth, silk gland weight, and cocoon quality, whereas higher concentrations negatively affected these parameters. These findings suggest that microbial supplementation can optimize insect growth and silk yield through improved nutrient assimilation and metabolic efficiency.

At a broader agricultural scale, insect-based feed for livestock presents a promising strategy for reducing the environmental impact of ruminant farming (contribution 10). Insects offer a renewable, high-protein alternative to conventional feeds such as soybean meal and fishmeal. Importantly, such feeds have the potential to lower methane emissions, a major contributor to agricultural greenhouse gases. However, large-scale implementation remains limited by regulatory uncertainty and the need for further research into feed safety, scalability, and economic feasibility (contribution 10).

Collectively, these studies underscore the multifaceted potential of insects across the food and feed continuum. From enhancing the nutritional and technological properties of human foods to improving livestock efficiency and reducing environmental pressures, insect-based innovations represent a crucial step toward achieving sustainable and resilient agri-food systems.

- **Vector biology, pathogen transmission, and medical and forensic entomology (research addressing medically important arthropods, their role in pathogen transmission, host–parasite interactions, factors influencing vector–host dynamics and human health the integration of molecular techniques into forensic entomology, improving species identification and post-mortem interval estimation).**

Several contributions address medically important arthropods and the integration of molecular approaches into public health and forensic science.

Kiewra et al. (contribution 11) conducted a multi-regional study in Poland and the Czech Republic revealing substantial differences in pathogen prevalence between countries and habitat types, with *Borrelia* infections being more frequent in ticks from Poland and from protected areas than in those from urban environments. Such findings highlight the complex interplay of ecological and environmental factors shaping pathogen circulation and underscore the importance of region-specific public health surveillance.

Bartosik et al. (contribution 12) report a clinical case of tick-bite granuloma following incomplete removal of an *Ixodes. ricinus* female demonstrating that localized inflammatory reactions can occur even after a short feeding period, emphasizing the need for public education on safe tick removal and post-bite monitoring. Vector–host interactions extend beyond ticks, as illustrated by studies on mosquito behavior and human physiological variability. Moreno-Gómez et al. (contribution 13) investigate how the menstrual cycle affects mosquito attraction and repellent efficacy, with higher landing rates and shorter repellent protection times during ovulation. These findings indicate that endogenous hormonal changes can modulate human attractiveness to mosquitoes, offering new insight into within-individual variability in biting risk and its implications for vector control strategies.

Scieuzo et al. (contribution 14) provide evidence on how molecular biology continues to expand the analytical power of entomology, particularly in forensic science. DNA-based techniques such as RAPD, RFLP, and mitochondrial sequencing, along with emerging tools like gene expression profiling and entomotoxicological analysis, have revolutionized the ability to identify insect species, estimate post-mortem intervals, and even retrieve human DNA from insect tissues. By strengthening the precision and evidentiary reliability of entomological data, molecular methods have become indispensable in modern forensic investigations.

Taken together, these studies indicate the broad spectrum of arthropod research, from disease ecology and host–vector interactions to forensic applications of molecular biology and underscore the central role of entomological and molecular approaches in improving human health, advancing diagnostic tools, and supporting evidence-based legal investigations.
